# Persistent Angina With Nonobstructive Coronary Artery Disease: Unmasking Coronary Microvascular Dysfunction Post-Oncotherapy

**DOI:** 10.1016/j.jaccas.2025.103795

**Published:** 2025-07-03

**Authors:** Philip Macanovic, Kevin Cheng, Anantharaman Ramasamy, John Baksi, Ranil de Silva

**Affiliations:** a Royal Brompton Hospital, Guy’s and St Thomas’ NHS Foundation Trust, Chelsea, London, United Kingdom; b National Heart and Lung Institute, Imperial College London, London, United Kingdom

**Keywords:** angina with nonobstructive coronary arteries, cancer, cardiac magnetic resonance, coronary microvascular dysfunction, Hodgkin lymphoma

## Abstract

**Background:**

Coronary microvascular dysfunction (CMD) is frequently the cause of patients presenting with angina with nonobstructed coronary arteries and may have increased prevalence in cancer survivors.

**Case Summary:**

We report a case of refractory angina in a survivor of Hodgkin lymphoma, previously treated with radiotherapy and chemotherapy. After demonstrating unobstructed epicardial arteries, advanced diagnostics including quantitative stress perfusion cardiac magnetic resonance and invasive coronary physiology testing, revealed CMD as a likely sequela of oncotherapy and informed initiation of mechanism-specific treatment.

**Discussion:**

CMD may represent an under-recognized cardiotoxicity of oncotherapy. This case highlights the value of formal, multimodal coronary microvascular function assessment to identify CMD and implement stratified management. Future systematic evaluation investigating the relationship and underlying mechanisms of oncotherapy in CMD is warranted.

**Take-Home Message:**

In cancer survivors presenting with angina with nonobstructed coronary arteries, clinicians should have a high index of suspicion for CMD and consider systematic evaluation to guide stratified treatment.

## Visual Summary

**Figure:**
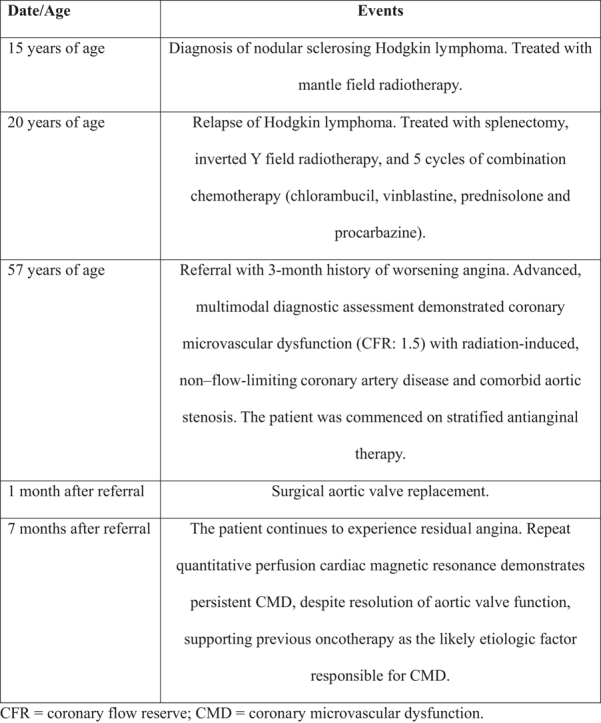

## History of Presentation

A 57-year-old man was referred with a 3-month history of worsening angina associated with dyspnea on exertion and reduced exercise tolerance. On examination, an ejection systolic murmur was identified, loudest at the right upper sternal border, radiating to the right carotid arteries.

**Take-Home Messages**

• Clinicians should maintain a high index of suspicion for coronary microvascular dysfunction in cancer survivors presenting with ANOCA, particularly in those exposed to mediastinal radiotherapy.
• Multimodal diagnostic strategies combining advanced imaging and invasive techniques enable accurate diagnosis and characterization of the underlying mechanisms of ischemia to guide personalized treatment stratification.

## Past Medical History

The patient had a background of nodular sclerosing Hodgkin lymphoma diagnosed 15 years of age. Treated initially with mantle field radiotherapy, he subsequently underwent splenectomy, inverted Y field radiotherapy, and 5 cycles of combination chemotherapy (chlorambucil, vinblastine, prednisolone, and procarbazine) at 20 years of age for recurrence.

## Differential Diagnosis

The differential diagnoses considered included flow-limiting epicardial coronary artery disease (CAD), aortic stenosis (AS), or angina with nonobstructive coronary arteries (ANOCA).

## Investigations

A 12-lead electrocardiogram and high-sensitivity troponin T were unremarkable. Treadmill exercise stress echocardiography showed no inducible regional wall motion abnormalities with normal left ventricular (LV) ejection fraction. Severe AS (mean rest and stress gradient: 22 and 42 mm Hg, respectively; peak stress velocity: 4 m/s; aortic valve area: 0.9 cm^2^) was confirmed. The scan was stopped early due to development of angina with the patient achieving 84% maximum heart rate at a workload of 4.5 METs, developing 1.5-mm horizontal ST-segment depression in the inferolateral (II, III, aVF, V_2_ to V_6_) leads.

**Figure 1:**
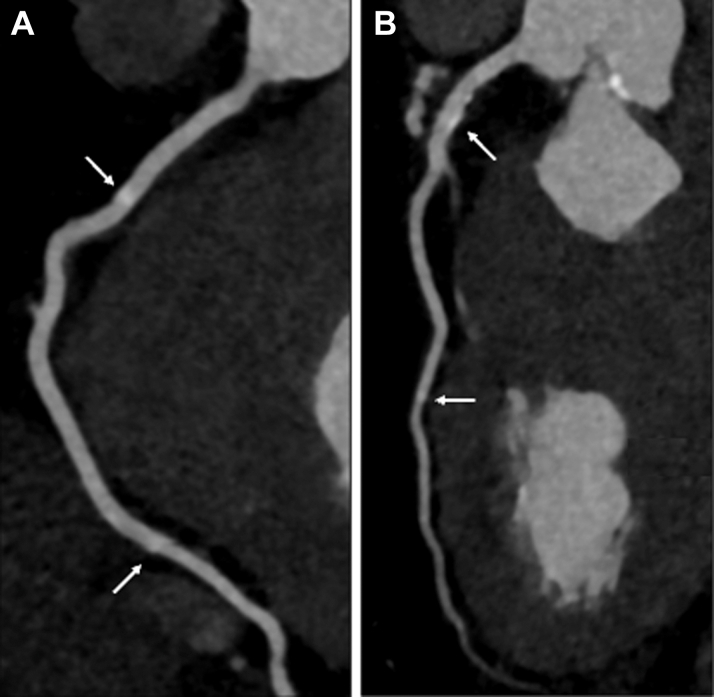
Computed Tomography Coronary Angiography Contrast enhanced computed tomography of the right (A) and left anterior descending (B) coronary arteries revealing radiation-induced calcific atheroma (arrows) and no obstructing coronary artery disease.

Given the clinical suspicion of significant epicardial CAD, a computed tomography coronary angiogram was performed demonstrating calcific but non–flow-limiting atheromatous disease (Figure 1).

**Figure 2:**
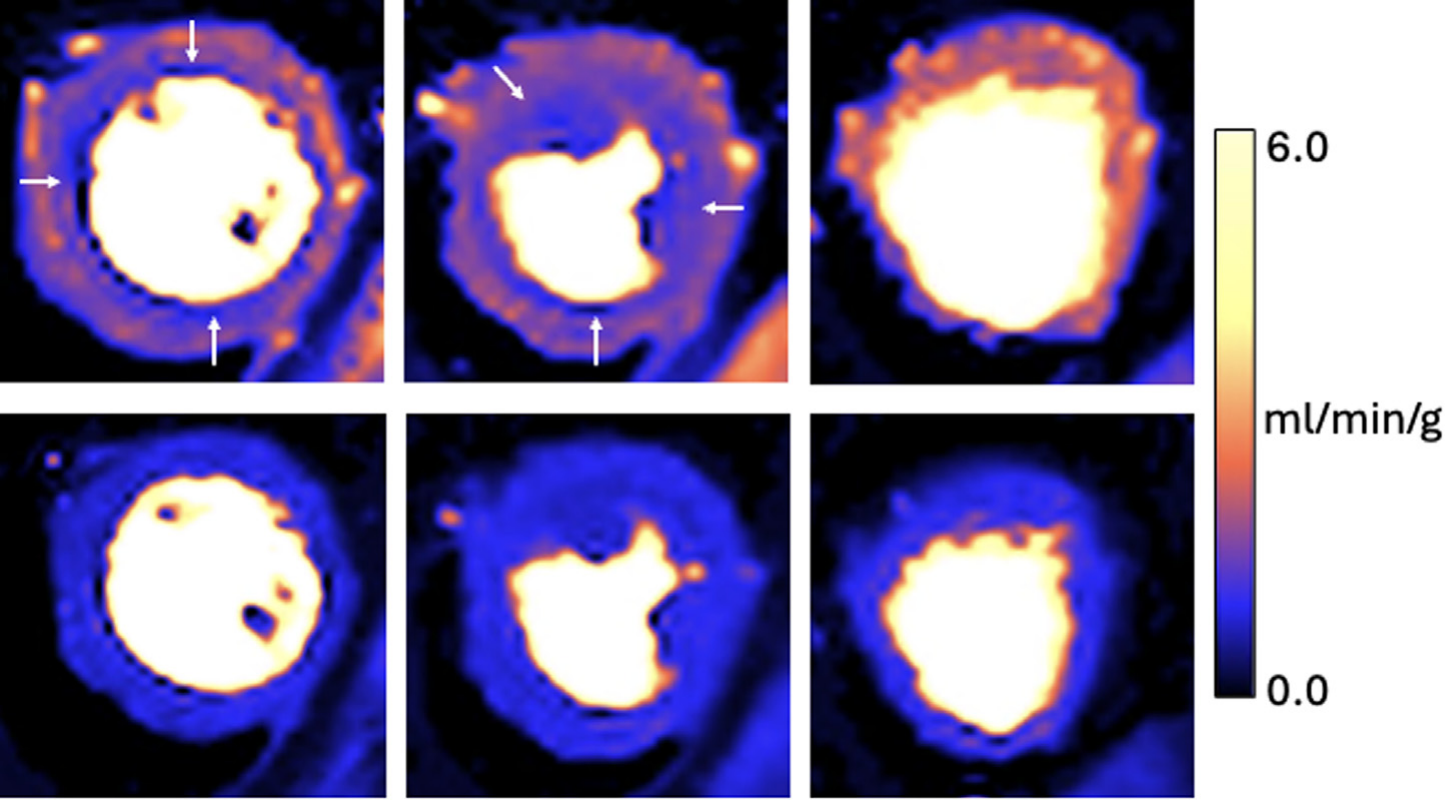
Quantitative Perfusion Cardiac Magnetic Resonance Fully automated inline quantitative perfusion cardiac magnetic resonance. Pixel-wise myocardial blood flow maps in basal, mid, and apical short-axis views are presented during adenosine-induced stress (top) and at rest (bottom). Significant inducible circumferential subendocardial hypoperfusion (open arrows) is demonstrated in the basal and mid-ventricular segments.

To further investigate the mechanism of angina, a quantitative perfusion cardiac magnetic resonance (CMR) study (Siemens 3-T Magnetom Vida) was performed using intravenous adenosine (180 μg/kg/min) as a pharmacologic vasodilator stressor as described previously.^1^ Normal biventricular volumes and ejection fraction were observed, with no evidence of LV hypertrophy. Global rest myocardial blood flow (MBF) was normal (0.9 mL/min/g). During hyperemia, significant inducible circumferential subendocardial hypoperfusion was observed with reduced global stress MBF (1.63 mL/min/g). Global myocardial perfusion reserve, calculated as the ratio of stress MBF over rest MBF, was reduced at 1.81 (abnormal: <2.2),^2^ particularly in the subendocardium (Figure 2). In the absence of obstructive epicardial coronary disease, these findings suggest coronary microvascular dysfunction (CMD). The patient was commenced on stratified antianginal therapy with ranolazine.

An invasive coronary angiogram with both epicardial and microvascular physiology assessment was performed. This confirmed non–flow-limiting epicardial CAD in the left anterior descending (LAD) artery by both nonhyperemic (resting full cycle ratio: 0.95; normal: >0.89) and hyperemic (fractional flow reserve: 0.89; normal: >0.80) metrics.

**Figure 3:**
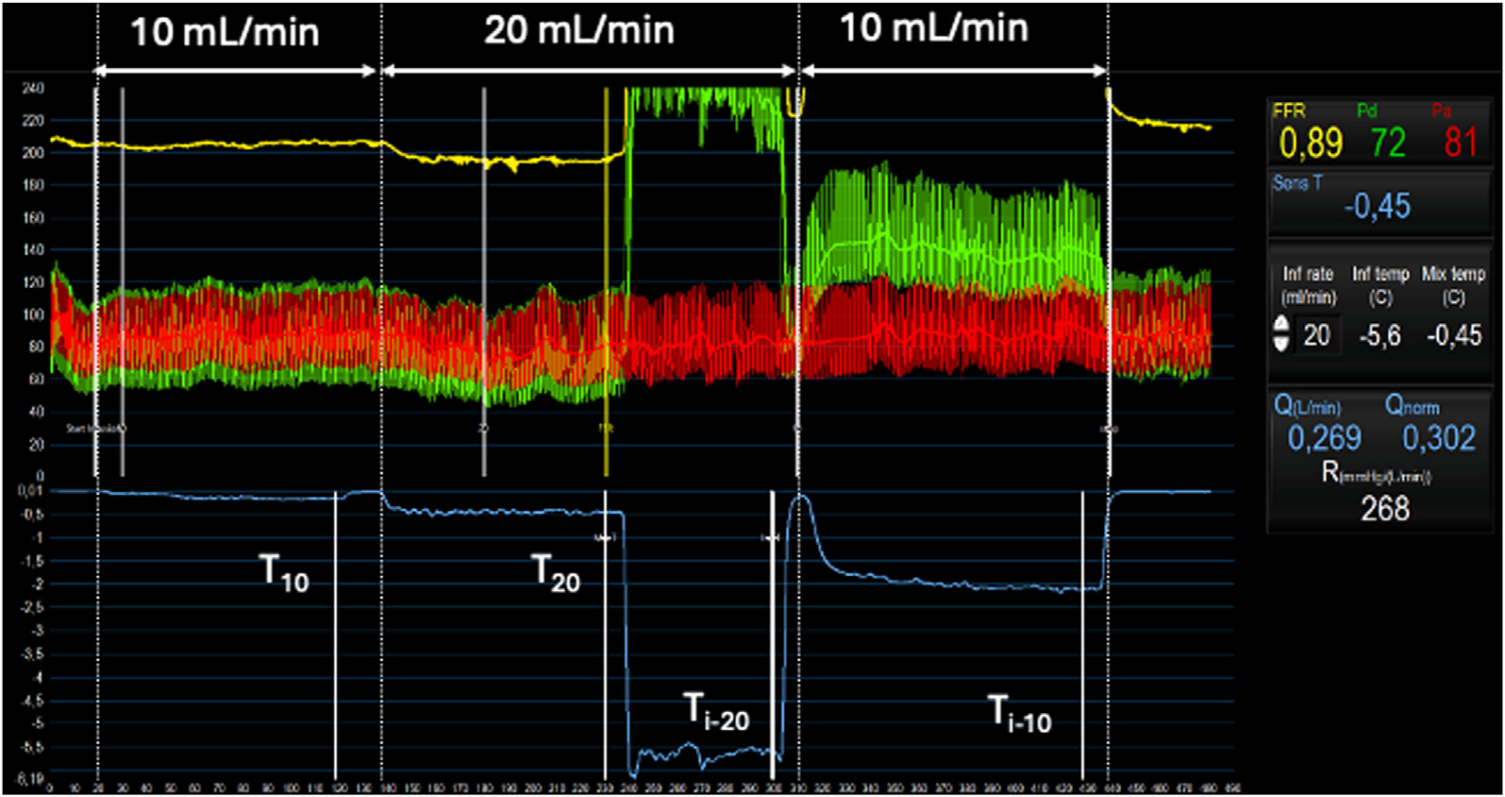
Continuous Thermodilution Coronary Function Assessment Simultaneous recordings of phasic and mean aortic pressure (red tracing), distal coronary pressure (green tracing), fractional flow reserve (yellow tracing), and temperature (blue tracing) were measured during continuous thermodilution assessment of the left anterior descending coronary artery (LAD). Room temperature saline was initially infused into the proximal LAD via a microcatheter at a rate of 10 ml/min and the distal intracoronary temperature change was measured (T_10_). Next, the infusion rate was increased to 20 mL/min and the distal intracoronary temperature change was measured (T_20_). The dual pressure and temperature sensor coronary guidewire was then withdrawn to the tip of the infusion catheter and the infusion temperature was measured (T_i-20_). The infusion rate was decreased to 10 mL/min and the infusion temperature was then measured (T_i-10_). These parameters were used to calculate indices of coronary microvascular function: flow, coronary flow reserve, absolute coronary resistance, and microvascular resistance reserve.

Next, we performed continuous thermodilution in the LAD (Figure 3). This technique has greater reproducibility compared with bolus thermodilution and enables measurement of absolute coronary blood flow and microvascular resistance.^3^ A dual pressure and temperature sensor coronary guidewire, PressureWire X (Abbott Vascular Inc) was advanced into the distal LAD. Then a 2.52-F infusion catheter (RayFlow; Hexacath) was advanced over the guidewire into the proximal LAD to deliver a continuous infusion of room temperature normal saline. A steady-state continuous infusion at 10 and 20 mL/min was administered to measure resting and hyperemic flow, and resistance. We measured significantly reduced coronary flow reserve (CFR) of 1.5 and microvascular resistance reserve of 1.83 in keeping with a structural endotype of CMD.

## Management

Alongside stratified antianginal therapy, the patient subsequently underwent uncomplicated surgical aortic valve replacement (SAVR) with 23-mm Inspiris Resilia bioprosthesis (Edwards Lifesciences).

## Outcome and Follow-Up

Postdischarge, the patient experienced improvement in dyspnea but continued to report residual angina. A repeat quantitative perfusion CMR was performed 6 months after SAVR which confirmed normal LV systolic and aortic valve bioprosthesis function. During hyperemia, inducible circumferential subendocardial hypoperfusion persisted with global myocardial perfusion reserve remaining reduced at 1.56, suggesting persistent CMD after SAVR. The results support previous oncotherapy as the etiologic factor responsible for CMD.

## Discussion

The population of patients undergoing cancer treatment is increasing in age and comorbidities. With the expanded use of oncology therapies that are known to promote endothelial dysfunction, hypertension, abnormal coronary vasomotion, and atherogenesis,^4^ an increasing number of cancer patients can be expected to present with acute and nonacute myocardial ischemic syndromes.^5^

### Oncotherapy and coronary microvascular dysfunction

Cardiotoxicity occurs in one-half of Hodgkin lymphoma patients treated with chemotherapy and radiotherapy, with increasing prevalence on account of improved survival rates.^5^ Although multiple cardiac pathologies have been documented in Hodgkin lymphoma, the impact on coronary microcirculation remains unestablished.^5^

Oncotherapy can induce microvascular dysfunction in other vascular beds.^6^^,^^7^ This case demonstrates that cardiovascular toxicities of oncotherapy for Hodgkin lymphoma may extend to coronary microcirculation and potentially contribute to increased morbidity and adverse cardiovascular outcomes in survivors of Hodgkin lymphoma after treatment.

Mantle field mediastinal radiotherapy delivers high-dose incidental cardiac irradiation.^5^ The increase in cardiovascular disease in Hodgkin lymphoma survivors is dose-dependent.^5^ Several underlying mechanisms have been proposed, including myocardial fibrosis, reduced capillary density, and endothelial dysfunction,^4^ which may all contribute to CMD. Reactive oxygen species generated by radiotherapy may be key causal mediators by eliciting intracellular oxidative damage and activating transforming growth factor-β and nuclear factor kappa B.^4^ These pathways amplify reactive oxygen species generation through a positive feedback loop and promote downstream cytokine production. The resultant chronic inflammation and damage lead to collagen deposition, myocardial remodeling, and decreased nitric oxide bioavailability.^4^ Separately, in patients receiving chest radiotherapy for breast cancer, these effects manifest as impaired endothelium-dependent cutaneous microvascular vasoreactivity^7^ and may contribute in part to CMD demonstrated in this case.

The treatment of Hodgkin lymphoma in this patient preceded the incorporation of anthracyclines into standard therapy. These drugs are largely responsible for the chemotherapy-induced cardiotoxicities identified in Hodgkin lymphoma patients.^5^ Doxorubicin can cause progressive and irreversible damage to the coronary microcirculation through tunica intima hyperplasia, myofibroblast proliferation, collagen accumulation, and vasomotion defects.^6^ The increased uptake of anthracyclines in modern Hodgkin lymphoma therapy regimens and its use in other cancers^4^ present another pathway for potential CMD development in patients receiving this drug as part of their cancer treatment.

A case series undertaking coronary microvascular testing using bolus thermodilution in 4 patients has identified both functional and structural endotypes of CMD after oncotherapy.^8^ Bolus thermodilution is limited by overestimation of CFR, suboptimal reproducibility, and less robust correlation with other reference methods (eg, H_2_^15^O positron emission tomography).^3^ Systematic studies investigating causal links between oncotherapy and CMD are lacking and needed. Identification of the presence of the underlying mechanisms of ischemia, including CMD and its associated endotypes, offers the potential for treatment selection, appropriately stratified to the underlying mechanisms of ischemia in an individual patient.

### Noninvasive evaluation of CMD

Current guidelines position invasive coronary physiology as the gold standard for assessment of ANOCA.^9^ However, this case emphasizes the evolving role of contemporary noninvasive assessment of CMD. Ischemic changes during exercise electrocardiogram, as observed in this case, were recently demonstrated to be highly specific for CMD and endothelial dysfunction in ANOCA patients.^10^ Quantitative perfusion CMR can identify CMD, including subendocardial hypoperfusion,^2^ while simultaneously providing information of other features of cardiotoxicity including LV dysfunction, LV remodeling, cardiac fibrosis, and edema. CMR is an attractive noninvasive ionizing radiation-free modality for evaluating and monitoring cancer patients at risk of cardiotoxicity.

### AS and CMD

CMD has been associated with AS through several mechanisms. Initial changes occur in the coronary microvasculature due to increased LV stroke work, resulting in an exhausted vasodilatory capacity.^1^ As AS severity progresses, increased LV diastolic pressures with associated LV hypertrophy, fibrosis arteriolar remodeling, and capillary rarefaction can lead to a structural endotype of CMD.^1^ These changes can manifest as an impaired CFR which can improve within 6 to 12 months after aortic valve replacement.^1^ In the current report, there was no evidence of LVH and CMD persisted after SAVR, suggesting an alternate etiology for CMD rather than AS.

## Conclusions

In cancer survivors presenting with ANOCA, clinicians should have a high index of suspicion for CMD as a consequence of oncotherapy, and consider systematic evaluation to establish the diagnosis and guide stratified treatment. Noninvasive and invasive modalities are available for the evaluation of CMD, as demonstrated in this case, which enables stratified, personalized therapy. Future systematic evaluations of the effects of oncologic therapies on coronary microcirculatory function in addition to other forms of cardiotoxicity are warranted.

**Visual Summary:** Timeline of the Case

Date/Age	Events
15 years of age	Diagnosis of nodular sclerosing Hodgkin lymphoma. Treated with mantle field radiotherapy.
20 years of age	Relapse of Hodgkin lymphoma. Treated with splenectomy, inverted Y field radiotherapy, and 5 cycles of combination chemotherapy (chlorambucil, vinblastine, prednisolone and procarbazine).
57 years of age	Referral with 3-month history of worsening angina. Advanced, multimodal diagnostic assessment demonstrated coronary microvascular dysfunction (CFR: 1.5) with radiation-induced, non–flow-limiting coronary artery disease and comorbid aortic stenosis. The patient was commenced on stratified antianginal therapy.
1 month after referral	Surgical aortic valve replacement.
7 months after referral	The patient continues to experience residual angina. Repeat quantitative perfusion cardiac magnetic resonance demonstrates persistent CMD, despite resolution of aortic valve function, supporting previous oncotherapy as the likely etiologic factor responsible for CMD.

CFR = coronary flow reserve; CMD = coronary microvascular dysfunction.

## Funding Support and Author Disclosures

The authors have reported that they have no relationships relevant to the contents of this paper to disclose.
